# Spi-1, Fli-1 and Fli-3 (miR-17-92) Oncogenes Contribute to a Single Oncogenic Network Controlling Cell Proliferation in Friend Erythroleukemia

**DOI:** 10.1371/journal.pone.0046799

**Published:** 2012-10-08

**Authors:** Samer Kayali, Guillaume Giraud, François Morlé, Boris Guyot

**Affiliations:** 1 CGPhiMC, CNRS UMR5534, Université Claude Bernard Lyon1, Lyon, France; University of Hong Kong, Hong Kong

## Abstract

Clonal erythroleukemia developing in susceptible mice infected by Friend virus complex are associated with highly recurrent proviral insertions at one of three loci called Spi-1, Fli-1 or Fli-3, leading to deregulated expression of oncogenic Spi-1 or Fli-1 transcription factors or miR-17-92 miRNA cluster, respectively. Deregulated expression of each of these three oncogenes has been independently shown to contribute to cell proliferation of erythroleukemic clones. Previous studies showed a close relationship between Spi-1 and Fli-1, which belong to the same ETS family, Spi-1 activating fli-1 gene, and both Spi-1 and Fli-1 activating multiple common target genes involved in ribosome biogenesis. In this study, we demonstrated that Spi-1 and Fli-1 are also involved in direct miR-17-92 transcriptional activation through their binding to a conserved ETS binding site in its promoter. Moreover, we demonstrated that physiological re-expression of exogenous miR-17 and miR-20a are able to partially rescue the proliferation loss induced by Fli-1 knock-down and identified HBP1 as a target of these miRNA in erythroleukemic cells. These results establish that three of the most recurrently activated oncogenes in Friend erythroleukemia are actually involved in a same oncogenic network controlling cell proliferation. The putative contribution of a similar ETS-miR-17-92 network module in other normal or pathological proliferative contexts is discussed.

## Introduction

Despite the high number and heterogeneity of mutations observed between tumors, a few recurrent oncogenic networks usually drive proliferation and survival of most individual tumors derived from a given cell type. It has recently been shown that in a given tumor, driver mutations mostly impact only one gene in each oncogenic network and that looking for mutually exclusive mutations actually allows the identification of these networks [1]. Identifying and understanding such new oncogenic networks remains an interesting challenge that should help the rational design of new therapies.

Murine erythroleukemia induced by Friend viruses provides an interesting model of oncogenesis driven by random proviral integrations and allow the exploration of such oncogenic networks [2], [3]. Indeed, most of these clonal erythroleukemia are associated with one of three recurrent proviral insertions leading to *spi-1*, *fli-1* [4] [4] or *fli-3* genes [5] deregulated expression.

*Spi-1* and *fli-1* encode two different transcription factors, Spi-1/PU.1 and Fli-1, which belong to the same ETS family. The ETS family comprises more than 30 members characterized by a conserved DNA binding domain allowing their binding to a common minimal GGAA motif [6]. Several studies already established that Spi-1 or Fli-1 over-expression actively contributes to the proliferation, survival and inhibition of differentiation of Friend erythroleukemic cells [7]–[11]. Among many specific mechanisms identified, Spi-1 and Fli-1 contribute to the inhibition of erythroid differentiation by functional antagonisms against GATA-1 [12] or EKLF [13], respectively. Fli-1 is a direct activator of the *bcl2* and *mdm2* genes [14], [15] and a repressor of *ship1* [16], whereas Spi-1 is a direct activator of the cyclin-dependent kinase *cdk6* [17]. We also showed that Spi-1 directly activates *fli-1* expression in all erythroleukemic cells harboring an activated *spi-1* locus [7]. Given the recurrent activation of these two factors belonging to the same ETS family, we worked on the hypothesis that they might contribute to erythroleukemia through the deregulation of common target genes. In agreement with this possibility, we recently established that both Spi-1 and Fli-1, like many other ETS members, share several direct common target genes including a large proportion of genes involved in ribosome biogenesis [18].

*fli-3* encodes the well known oncogenic miRNA cluster miR-17-92. This miR-17-92 cluster comprises six miRNAs that can be grouped into four sub-families based on their seed sequence (miR-17 and miR-20a, miR-18a, miR-19a and b and miR-92a) [19]. Following the first observation of frequent miR-17-92 over-expression in B-cell lymphomas [20], it has been experimentally demonstrated that its over-expression can indeed accelerate development of B-cell lymphoma initiated by Myc deregulation [21], [22]. Since then, miR-17-92 deregulation has been documented in many different cancers and its functional implication in stimulation of cell proliferation and survival has been demonstrated in many different cell contexts [23]–[26] including in the factor-dependent Friend erythroleukemic cell line HB60 harboring an activated *spi-1* locus [5]. Except for miR-18, the oncogenic contributions of miR-17/20a, miR-19a/b and miR-92 have all been demonstrated and several functional targets identified, including E2F1, PTEN and BIM1 [26]. Intriguingly however, miR-17/20a have also been reported to behave as an anti-oncogene [27]–[29] but the cell context differences that determine their oncogenic versus anti-oncogenic properties remain poorly understood.

The present study aimed to investigate whether miR-17-92 could be a functional and direct target of Spi-1 and Fli-1 in Friend erythroleukemia. We found that miR-17-92 is indeed expressed in two erythroleukemic cell lines harboring either a *spi-1* or *fli-1* activated locus and that its expression decreases following Spi-1 and/or Fli-1 knock-down. We found in vitro that full miR-17-92 promoter activity is dependent on a conserved ETS binding site and that Spi-1 and Fli-1 bind this promoter in vivo. We further demonstrated that loss of proliferation induced by Fli-1 knockdown can be at least partially rescued by exogenous whole miR-17-92 cluster re-expression or by miR-17 and miR-20a only re-expression. Altogether, these results indicate that the three most frequently activated oncogenes in Friend erythroleukemia are linked into a single oncogenic network contributing to cell proliferation.

## Materials and Methods

### Cell lines, culture, and transfection

Mouse erythroleukemic cell line 745A (kindly provided by Dr F Moreau-Gachelin, Curie Institute, France), has been originally established from mice infected by Friend virus complex (composed of defective SFFV and helper F-MuLV viruses) [30], [31]. 745A cells harbor SFFV proviral insertion at *spi-1* locus responsible for deregulated production of Spi-1/PU.1 transcription factor leading in turn to *fli-1* gene activation and deregulated production of Fli-1 transcription factor [7]. Mouse erythroleukemic cell line NN10 (kindly provided by Dr F Wendling, Gustave Roussy Institute, France) has been established from newborn mice infected by helper F-MuLV virus only [32]. NN10 cells harbor F-MuLV proviral insertion at *fli-1* locus responsible for deregulated production of Fli-1. Clone#44 and NN10#5 has been derived from parental 745A and NN10 cells respectively through the establishment of Dox-inducible shRNA expression allowing conditional Fli-1 knock-down as previously described [18]. Clones NN10#17-92a and #17-92b expressing inducible exogenous miR-17-92 and clone NN10#17-20 expressing inducible exogenous miR-17 and miR-20a were derived from NN10#5 cells after transfection with expression vector pcDNA4/TO (Invitrogen, Cergy Pontoise, France) carrying the corresponding miRNA coding regions followed by zeocin selection. All cell clones were cultured at 37°C under 5% CO_2_ in a humidified incubator and in Iscove's modified Dulbecco's medium (PAA Laboratories, Les Mureaux, France) supplemented with 10% fetal calf serum (PAA Laboratories) and antibiotics. Exogenous miRNA and/or Fli-1 shRNA productions were induced by adding 100 ng/ml of Doxycycline (Dox) (Clontech, Saint-Germain-en-Laye, France). Terminal erythroid differentiation of 745A#44 cells associated with Spi-1 extinction was induced in the presence of 5 mM HMBA (hexamethylene bisacetamide, Sigma, Saint-Quentin Fallavier, France). Transfections were performed either by lipofection using FuGENE 6 (Roche, Meylan, France) or by nucleofection using the “cell line nucleofector Kit V” and program G-16 on a Nucleofector electroporation device (Lonza, Basel, Switzerland). Anti-miRs were purchased from Applied Biosystems. 20 pmol of anti-miRs were introduced into 2.10^5^ cells using a Nucleofector device and a “Cell line nucleofector kit V” using program G-16 (Lonza, Basel, Switzerland). siRNAs transfections were performed using the same protocol using 120 pmol of siRNAs and 2.10^5^ cells.

### Plasmid constructs

pcDNA4/17-92 and pcDNA4/17-20 were obtained by cloning PCR-amplified genomic DNA corresponding to the whole mouse miR-17-92 cluster or to the miR-17 and miR-20a coding segments downstream to the Dox-inducible CMV promoter in pcDNA4/TO plasmid (Invitrogen, Cergy Pontoise, France). pGL3-EBS reporter construct was obtained by cloning the mouse −645/+15 miR-17-92 cluster promoter region upstream to the luciferase coding region in pGL3basic (Promega, Charbonnières, France). To mutate the −78 EBS from CGGAAG to ACGCGT in pGL3-EBSmut, we first PCR amplified from pGL3-EBS the DNA sequence immediately upstream of the −78 EBS with (−645/+15 promoter F) and (−78 EBS mutagenesis UE) primers. We similarly amplified the sequence downstream of the −78 EBS with (−645/+15 promoter R) and (−78 EBS mutagenesis DE) primers. Both PCR products were digested by Mlu1 and Xho1 and ligated in the Xho1 site of the pGL3basic vector. Sequences of primers used for these plasmid constructions are given in Table S1.

### Western blot analyses

Western blot analyses were performed on total cell lysates as previously described [18] using the following antibodies: anti-Fli-1 (C-19) (sc-356; Santa Cruz), anti-actin (MAB1501; Millipore), anti Spi-1 (T-21) (sc-352; Santa Cruz), anti-E2F1 (C-20) (sc-193; Santa Cruz), anti-p21 (C-19) (sc-397; Santa Cruz), anti-TxNip (K0205-3; MBL), anti PTEN (#9559; Cell Signaling), anti-cMyc (9E10) (sc-40; Santa Cruz) and anti-Hbp1 (#83402; Abcam).

### Gene set enrichment analyses

Microarray data from 745#44 cells untreated or treated for two days in the presence of Dox alone, HMBA alone or both Dox and HMBA have been previously obtained [18]. Lists of predicted miRNA targets were established from the miRWalk database (http://www.umm.uni-heidelberg.de/apps/zmf/mirwalk/) by compiling all mouse transcripts with 3′UTR harboring putative target sequences predicted by the three different algorithms miRanda, miRDB and miRWalk with a p value<0.05. Predicted targets lists were then used to perform gene set enrichment analyses (GSEA) by comparing the transcriptome of untreated or Dox+HMBA treated 745#44 cells (displaying the strongest differential in pri-miR-17-92 levels) using the GSEA software v2.0 (http://www.broadinstitute.org/gsea/index.jsp/) [33]. For each miRNA target lists displaying significant differential expression in response to HMBA and Dox, we retrospectively compared the mean relative expression levels of the corresponding leading edge subset of these lists between all four conditions (untreated, Dox alone, HMBA alone or Dox and HMBA). Given the very high similarity between miR-17 and miR-20a or miR-19a and miR-19b, this retrospective analysis was performed on the intersection of the leading edge subset of miR-17 and miR-20a or miR-19a and miR-19b, respectively. File containing all gene lists generated (excel file named “Genes lists GSEA”) as well as files containing transcriptome data used to perform GSEA (manip745.grp, manip745.chip and manip745.gct) are provided as supplementary data (Data S1 and S2).

### mRNA and miRNA quantification

For mRNA quantification, total RNA was extracted using the Rneasy PLUS minikit (Qiagen, Courtaboeuf, France) and reverse transcribed using a Quantitect reverse transcription kit (Qiagen) followed by quantitative PCR using a QuantiTect SYBR green PCR kit (Qiagen) and specific primers indicated in Table S1. mRNA specific signals were normalized to that of beta-actin mRNA. For miRNA quantification, total RNA was extracted using TriReagent (Sigma-Aldrich, Lyon, France) and reverse transcribed using a TaqMan reverse transcription kit (Applied Biosystems) followed by quantitative PCR performed using a TaqMan qPCR assay (miR-17, miR-20a, miR-18, miR-19a, miR-19b, miR-92a and U6 snoRNA specific kits: Applied Biosystems). miRNA specific signals were normalized using U6 RNA level.

### Chromatin immunoprecipitation assays (ChIP)

ChiP assays were performed exactly as previously described [18] using anti-Spi-1 (sc-352; Santa Cruz), anti-Fli-1 (sc-356; Santa-Cruz) and anti-UBC9 (sc-10759; Santa Cruz) followed by real-time PCR using a SYBR green PCR kit and specific primers indicated in Table S1.

### Promoter assays

NN10#5 cells (2×10^5^) were co-transfected using Fugene6 (Roche, Meylan, France) with a fixed amount of a β-galactosidase expressing vector and constant molar amounts of pGL3, pGL3-ETS or pGL3-EBSmut luciferase reporters plasmids while keeping the total amount of transfected DNA constant to 5 µg. After 24 hours, cells lysates were assayed for luciferase and β-galactosidase activities by chemiluminescence using the luciferase high sensitivity reporter gene assay and the β-galactosidase reporter gene assay kits (Roche, Meylan, France). Luciferase activities were normalized to β-galactosidase activities.

## Results

### Spi-1 and Fli-1 directly activate miR-17-92 expression in 745A#44 cells

**Figure 1:**
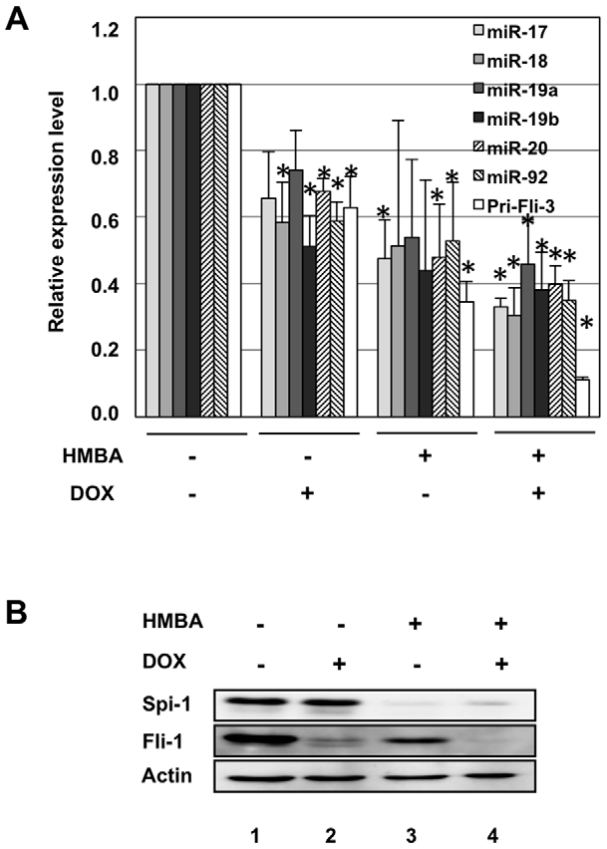
miR-17-92 expression correlates with ETS transcription factors Spi-1 and Fli-1 decrease during 745A#44 cells differentiation. A: As indicated, 745A#44 cells have been treated for two days with or without Dox to induce Fli-1 knockdown and for two other days in the same conditions with or without addition of 5 mM HMBA to induce differentiation associated with Spi-1 extinction. Pri-miRNA-17-92 transcript and mature miRNAs levels were then determined by semi-quantitative real-time RT-PCR using actin mRNA or U6 RNA as reference, respectively. The upper panel shows relative transcripts levels following Dox and/or HMBA treatment normalized to untreated cells levels (means and standard deviations from 3 independent experiments). Asterisks show significant differences compared to the untreated condition (t-test, p<0,05). B: Typical results of a Western blot analysis of Fli-1 and Spi-1 protein levels after treatments used in A.

**Figure 2:**
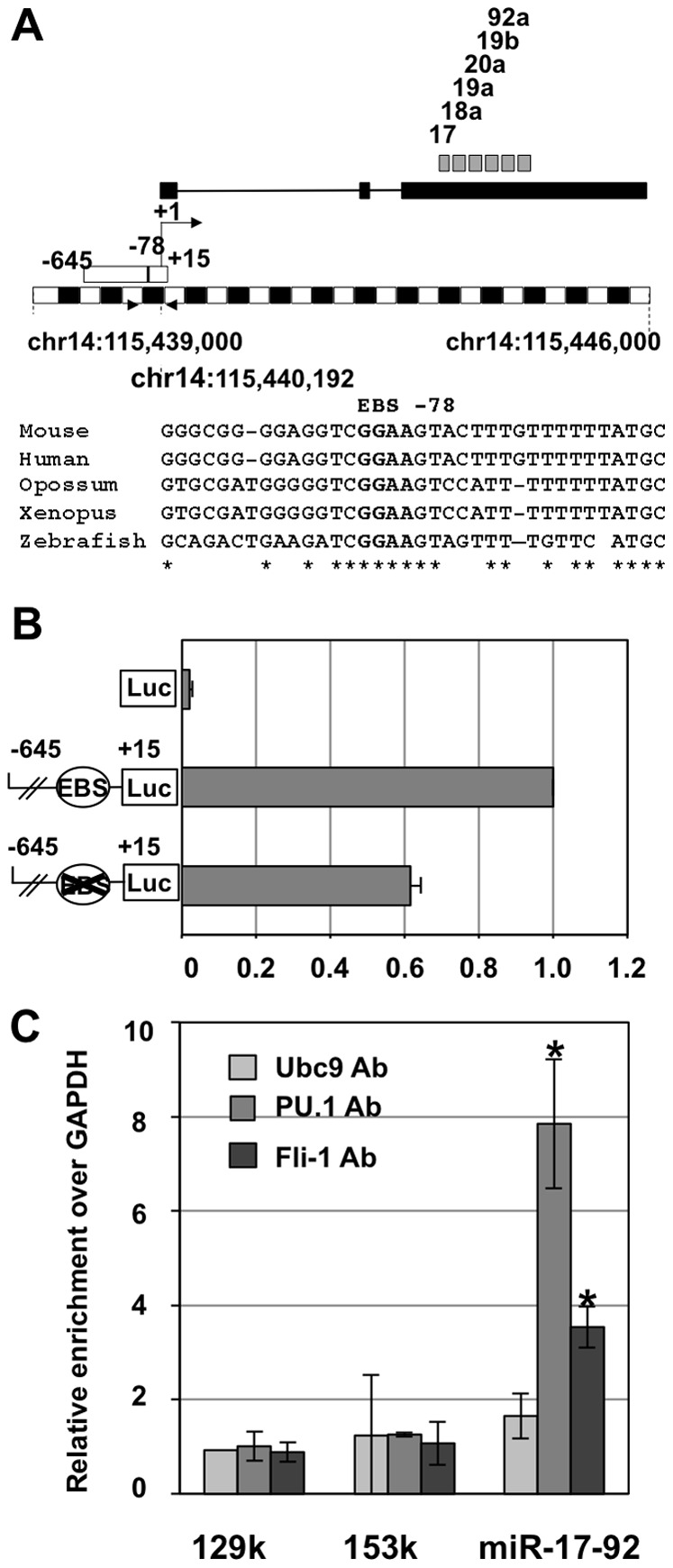
Spi-1 and Fli-1 directly activate miR-17-92 promoter in 745A#44 cells. A: Schematic map of the mouse chromosome 14: 115,439,000–115,445,000 interval harboring the miR-17-92 cluster and flanking regions. Blacks and white boxes drawn upper the scale (250 bp units) correspond to the putative miR-17-92 primary transcript and to the −645/+15 promoter region used in B, respectively. Locations of the 6 different mature miRNAs sequences of the cluster are indicated by grey boxes. Primers used for ChIP analyses of Spi-1 and Fli-1 binding are indicated by black triangles. Sequences alignment illustrates −78 EBS conservation in Human, Mouse, Opossum, Xenopus and Zebrafish genomes. B: Reporter gene assays showing promoter activity of the −645/+15 region and its partial dependency on the conserved −78 EBS. 745#44 cells were co-tranfected with pCMV-βGal and pGL3, pGL3-EBS or pGL3-EBSmut luciferase reporter constructs as indicated. Luciferase activities were normalized to β-galactosidase determined 24 h after transfection and normalized relative luciferase activities were expressed with respect to the wild type construct (means and standard deviations from 3 different experiments). C: Chromatin immunoprecipitation (ChIP) assays showing Spi-1 and Fli-1 binding on the endogenous miR-17-92 promoter in 745A#44 cells. ChiPs assays were performed using Spi-1, Fli-1 or UBC9 (negative control) antibodies followed by q-PCR of mir-17-92 promoter and three negative control regions: GAPDH promoter, 129k and 153k. Results are expressed as relative enrichments of the sequence of interest over the GAPDH promoter sequence normalized to the enrichment determined on 129k region with control UBC9 antibody (means and standard deviations from 3 independent experiments). Asterisks indicate significant increase in the enrichment of miR-17-92 compared to 153k control sequences obtained with Fli-1 and PU.1 antiobodies (p<0.05).

We previously established that Spi-1 and Fli-1, the two most frequently activated oncogenes in Friend erythroleukemia, display tight functional connections since Spi-1 directly activates the *fli-1* gene [7] and they both directly activate target genes involved in ribosome biogenesis [18]. These results prompted us to investigate if Spi-1 and Fli-1 could also be involved in the direct activation of *fli-3*, the third most commonly activated locus in Friend erythroleukemia, encoding the known miR-17-92 oncogenic miRNA cluster [5]. For this purpose, we used our previously described 745A#44 erythroleukemic cells model carrying an activated *Spi-1* locus and expressing Doxycyclin-inducible Fli-1 shRNA [18]. These cells can be induced to differentiate by the additive effect of HMBA (hexamethylene bisacetamide) and Doxycycline leading to Spi-1 extinction and shRNA mediated Fli-1 knockdown, respectively (Figure 1B). We found that successive decrease of Fli-1 by Dox (lane 2), Spi-1 by HMBA (lane 3) or of both Spi-1 and Fli-1 by combined Dox and HMBA treatments (lane 4) were associated with a progressive decrease in pri-miR-17-92 transcripts as well as all six mature miRNAs of the cluster (Figure 1A). Such a progressive decrease in pri-miR-17-92 transcript levels is compatible with the hypothesis of an additive contribution of Spi-1 and Fli-1 in miR-17-92 cluster transcriptional activation. Interestingly, we noticed a highly conserved ETS binding site located at position −78 (Figure 2A and 2B) in the known miR-17-92 promoter [21], [34]. Using luciferase reporter assays, we confirmed that the mouse −645/+15 promoter region indeed displayed a strong promoter activity that decreased by 40% after the −78 ETS binding site mutation (Figure 2B). In agreement with these results, ChIP assays performed on 745A#44 untreated cells showed an eight and three fold enrichment of the miR-17-92 promoter region compared to control GAPDH promoter region in chromatin immunoprecipitated with Spi-1 or Fli-1 specific antibodies, respectively. In contrast, chromatin immunoprecipitated with control UBC9 antibody showed no significant enrichment in miR-17-92 promoter (Figure 2C). These results altogether support the hypothesis that both Spi-1 and Fli-1 contribute to miR-17-92 cluster expression through a direct binding to its promoter, potentially at the functional and conserved ETS binding site we identified.

### Identification of miR-17/miR-20a and miR-19a/miR-19b signatures in the transcriptome of 745A#44 cells displaying decreased levels of miR-17-92 cluster

**Figure 3:**
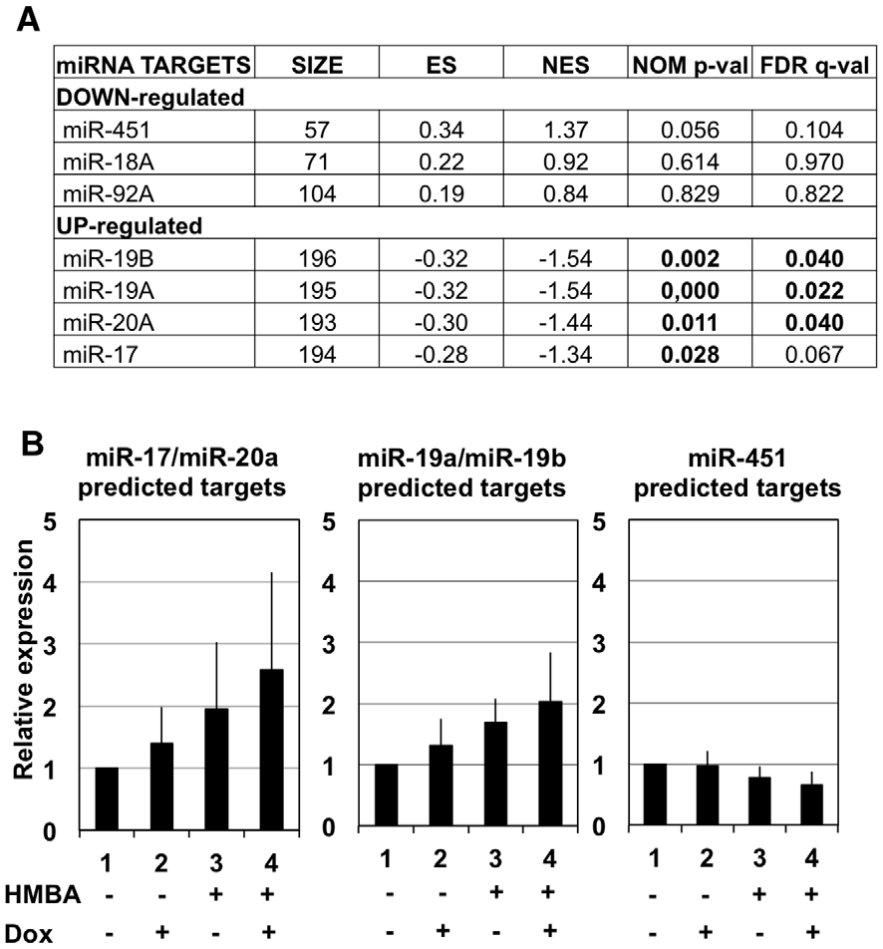
Up-regulation of miR-17-20a and 19a-19b predicted targets following Spi-1 and Fli-1 knockdown in 745A#44 cells. A: GSEA (Gene Set Enrichment Analysis) of predicted miRNA targets in 745A#44 cells treated for two days with both HMBA and Dox versus untreated cells. Lists of targets predicted by three different algorithms (miRanda, miRDB and miRWalk) were compiled for each miRNAs from miRWalk web site. The size column displays the total number of predicted targets used for the analysis, ES and NES indicate the standard and normalized enrichment scores respectively and the last two columns display the normalized p-values and false discovery q-values of the enrichments. Significant enrichments are indicated in bold. B: Mean relative expression profiles of the leading edge subsets of common predicted targets for miR-17/20a, miR-19a/19b and miR-451 identified by GSEA in response to progressive decrease of miR-17-92 expression or progressive increase of miR-451 induced by indicated combinations of HMBA and Dox treatment in 745A#44 cells.

In order to test the functionality of individual miRNA of the miR-17-92 cluster in 745A#44 cells, we checked if their transcriptome was significantly affected by miR-17-92 expression decrease induced by combined HMBA and Dox treatments. We addressed this question using Gene Set Enrichment Analysis using transcriptome data obtained from 745A#44 cells treated or not for two days with HMBA and Dox [18] and lists of miRNA targets predicted from 3′UTR analyses by three different algorithms (miRanda, miRDB and miRWalk) [35]. This analysis (Figure 3A) revealed that genes belonging to the predicted targets lists of miR-17, miR-20a, miR-19a and miR19b but not to those of miR-18a or miR-92a were significantly over-represented among genes up-regulated in response to HMBA and Dox (resulting in down regulation of miR-17-92 cluster). As a control, target genes of miR-451, which is up-regulated in response to HMBA [36], tend in contrast to be over-represented among down-regulated genes. We also compared mean expression levels of genes in the leading edge subsets of common miR-17/miR-20a, common miR-19a/miR-19b and miR-451 targets identified in response to HMBA in the other two conditions (+Dox only or +HMBA only) which are associated with intermediate pri-miR-17-92 expression levels (Figure 1). As expected from authentic target genes, variations in the levels of miR-17/miR-20a and of miR-19a/miR-19b targets were inversely correlated with the variations of pri-miR-17-92a levels (compare Figure 3B with pri-miR-17-92a and miRNAs profiles in Figure 1A). In summary, these analyses indicated that miR-17-92 cluster in 745A#44 cells is mainly involved in the down-regulation of predicted targets of miR-17/miR-20a and miR-19a/miR-19b rather than miR-18a or miR-92a.

### Fli-1 directly activates miR-17-92 expression in NN10#5 cells

**Figure 4:**
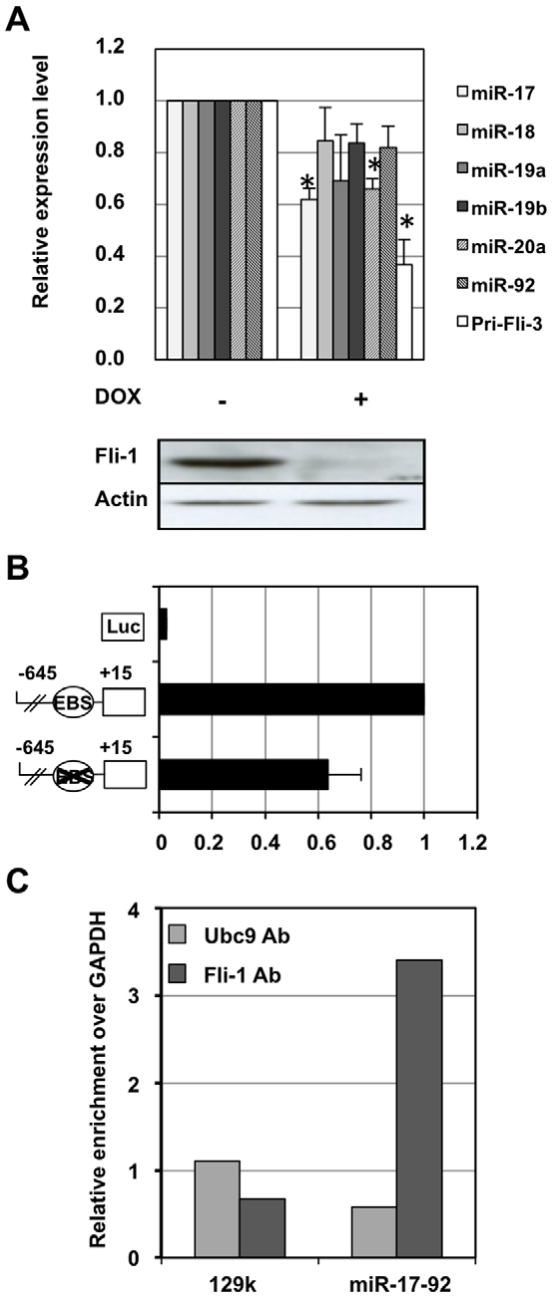
Fli-1 directly activates miR-17-92 promoter and contributes to miR-17 and miR-20a expression in NN10#5 cells. A: NN10#5 cells have been cultured for two days in absence or presence of Dox used to induce Fli-1 knock-down before analysis. Histogram shows the relative decreases of pri-miRNA-17-92 transcript and mature miRNA levels in treated versus untreated cells (means and standard deviations from 3 independent experiments) with significant (p<0.01) decreases indicated by asteriks. A western blot analysis confirming the strong decrease of Fli-1 protein in treated cells is shown under the histogram. B: Reporter gene assay showing promoter activity of the −645/+15 region and its partial dependency on the conserved −78 EBS. NN10#5 cells were co-transfected as indicated for 745#44 cells in Figure 2B. C: ChIP analyses performed in NN10#5 cells using Fli-1 or control UBC9 antibodies followed by q-PCR of miR-17-92 promoter and GAPDH or 129k negative control regions. Results are expressed as relative enrichments normalized to background enrichment determined on the GAPDH region (results from a single experiment).

The above results prompted us to ask if miR-17-92 cluster expression was also regulated by Fli-1 in erythroleukemic cells harboring a *fli-1* instead of *spi-1* proviral integration. We used as a model our previously described NN10#5 cells harboring an activated *fli-1* and a dox-inducible Fli-1 shRNA expression [18]. Dox treatment of NN10#5 led to a strong reduction of Fli-1 levels associated with a 60% decrease of pri-miR-17-92 transcript level and a 40% decrease of mature miR-17 and miR-20a levels (Figure 4A). Dox treatment was also associated with smaller and not statistically significant decreases of all other mature miRNAs. We then showed by reporter gene assay that the mouse −645/+15 promoter region displayed a strong promoter activity in NN10#5 cells that decreased by 40% following mutation of the −78 ETS binding site (Figure 4B). Moreover, ChiP assays confirmed Fli-1 recruitment on endogenous miR-17-92 promoter in NN10#5 cells as revealed by a 3.5 higher enrichment of the corresponding region over GAPDH control region in chromatin immunoprecipitated by Fli-1 antibody but not by control UBC9 antibody (Figure 4C). These results indicate that a contribution to the direct transcriptional activation of the miR-17-92 cluster by constitutively expressed ETS factors seems to be a common property of Friend erythroleukemic cells harboring either *spi-1* or *fli-1* proviral integrations.

### miR-17-92 functional implication in NN10#5 cells proliferation control downstream of fli-1

**Figure 5:**
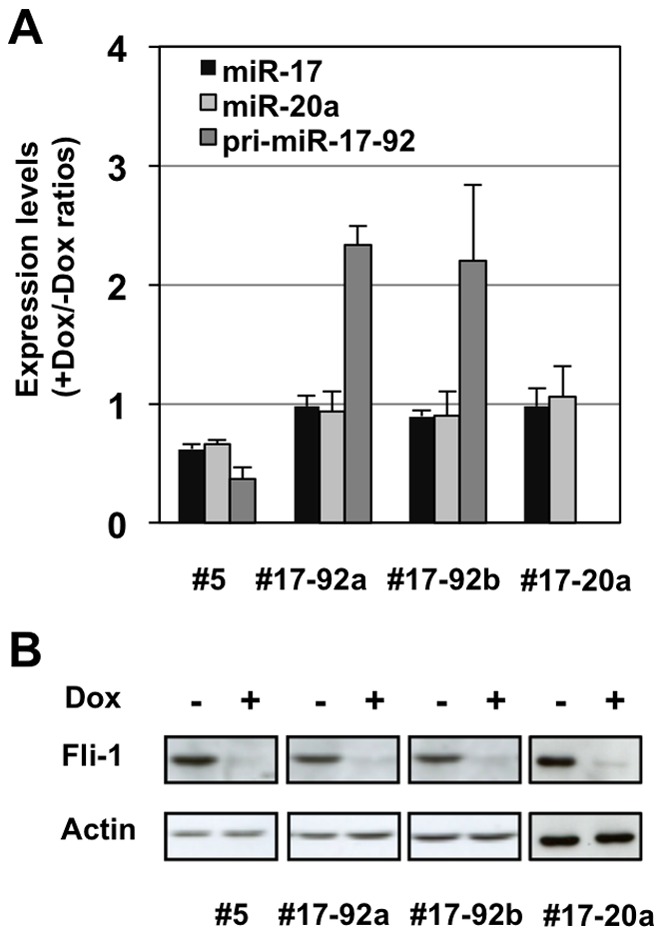
Restoration of initial levels of miR-17 and miR-20a following Dox-induced Fli-1 knockdown in NN10#5 cells. #17-92a, #17-92b or #17-20 cell clones have been derived from parental NN10#5 cells following stable transfection of a Dox-inducible expression cassettes of the whole miR-17-92 cluster or only miR-17-20a sub-cluster, respectively. A: Parental NN10#5 cells and their derivative clones #17-92a, #17-92b or #17-20 harboring inducible exogenous whole miR-17-92 miRNA cluster or miR-17-20a sub-cluster were cultured for two days in the presence or absence of Dox and the relative levels of pri-miR-17-92, miR-17 and miR-20a levels were determined by qRT-PCR as in Figure 4. Results are expressed as +Dox/-Dox ratios (means and standard deviations from 3 independent experiments). B: Fli-1 expression analysis by western blot in indicated cell clones after two days in presence or absence of Dox using β-actin signal as a loading control.

**Figure 6:**
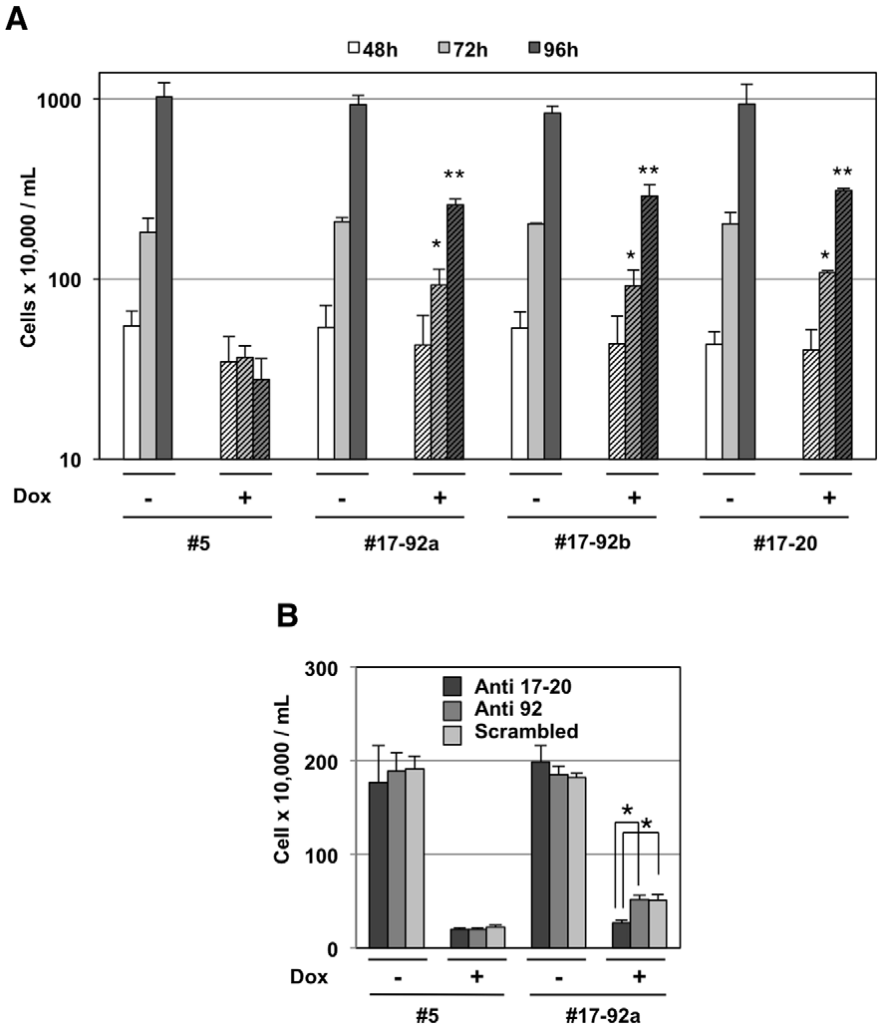
miR-17 and miR-20a are sufficient for partial rescue of Fli-1 knock-down induced cell proliferation arrest. A: Equal numbers of parental NN10#5 cells and of their three derivative clones #17-92a, #17-92b and #17-20 were cultured for 96 h in the presence or absence of Dox as indicated. Histogram shows cell concentrations (log scale) at 48 h, 72 h and 96 h time points, 48 h being the minimum Dox treatment time required for complete Fli-1 knockdown (mean and standard deviations from 3 independent experiments). Asterisks (p<0.05) and double asterisks (p<0.01) show significant differences between NN10#5 cells and derivatives in presence of Dox. B: Equal numbers of NN10#5 or #17-92a cells were transfected with control, anti-miR-92a or a mixture of anti-miR-17 and anti-miR-20a and cultured in the presence or absence of Dox. Cell proliferation was determined 72 h after transfection, the latest time point at which anti-miRs are still efficient. Mean and standard deviations of cell densities (n = 3) are shown (linear scale). Statistically significant differences are indicated by asterisks (p<0.01).

We previously established that Dox-induced Fli-1 knock down strongly decreased NN10#5 cells proliferation [18]. The above results raised the interesting possibility that Fli-1 critical contribution to cell proliferation could be mediated at least partly by the miR-17-92 cluster. To address this question, we first tried to maintain miR-17-92 expression in absence of Fli-1 in order to test if NN10#5 cells proliferation could be rescued during Dox treatment. For that purpose, we derived sub-clones from parental NN10#5 cells with a stable integration of a plasmid carrying the whole miR-17-92 cluster under the control of a Dox-inducible promoter. We thus identified clones #17-92a and #17-92b that maintained stable levels of mature miR-17 and miR-20a in the presence or absence of Dox (Figure 5A) while still displaying Dox-inducible Fli-1 knock down (Figure 5B). As previously reported [18] Dox treatment of parental NN0#5 cells abolishes their proliferation. In contrast, Dox treatment of #17-92a and #17-92b cells induced an only 2 fold decrease in proliferation rates (doubling time of 12 hours for NN10#5 and #17-92 clones without Dox versus 24 hours for #17-92a and #17-92b cells in presence of Dox) thus indicating a partial compensation of Fli-1 loss (Figure 6A). We also derived following the same strategy a single clone from 745#44 cells that maintains stable levels of pri-miR-17-92 and miR-20a after Fli-1 knockdown (Figure S1). However, unlike in NN10#5, restoration of pri-miR-17-92 and miR-20a levels in 745#44 cells did not rescue cell proliferation decrease induced by Fli-1 loss thus suggesting different parallel contributions of Fli-1 between the two cell lines. We then wanted to know which members of the miR-17-92 cluster were involved in NN10#5 cells proliferation. We decided to focus first on miR-17 and miR-20a which are the most significantly downregulated miRNA of the cluster after Dox treatment. In addition, miR-17 and miR-20a are two closely related miRNA which share a common seed sequence and have several well described targets. Using the same approach, we derived #17-20a cells allowing Dox-inducible expression of miR-17 and miR-20a only instead of the whole miR-17-92 cluster. Like in #17-92a and #17-92b, miR-17 and miR-20a levels remained roughly unaffected by Dox treatment in #17-20 cells (Figure 5A) while Fli-1 remained strongly down regulated (Figure 5B). Interestingly, when compared to parental NN10#5 cells, #17-20a cells displayed the same proliferation rescue in Dox as did #17-92 a and #17-92b cells (Figure 6A), thus indicating that combined miR-17 and miR-20a re-expression is sufficient to reproduce the effect of the whole miR-17-92 cluster. In a complementary approach, we repeated these proliferation measures between parental #5 and #17-92a cells with concomitant transfection of a miR-17 and miR-20a anti-miRs mixture, of miR-92 anti-miR or of control anti-miR. We used a miR-17 and miR-20a anti-miRs mixture since both miRNA share a common seed sequence and have essentially identical targets. Transfection of either miR-92 or control anti-miRs did not affect the partial proliferation rescue in #17-92 cells in presence of Dox (Figure 6B). In contrast, miR-17 and miR-20a anti-miRs transfection completely suppressed the proliferation rescue of #17-92a cells in the presence of Dox, thus confirming that exogenous miR-17-92 cluster contribution is strictly dependent on miR-17 and miR-20a function.

### Identification of HBP1 as a miR-17/miR-20a target in NN10#5 and 745A#44 cells

**Figure 7:**
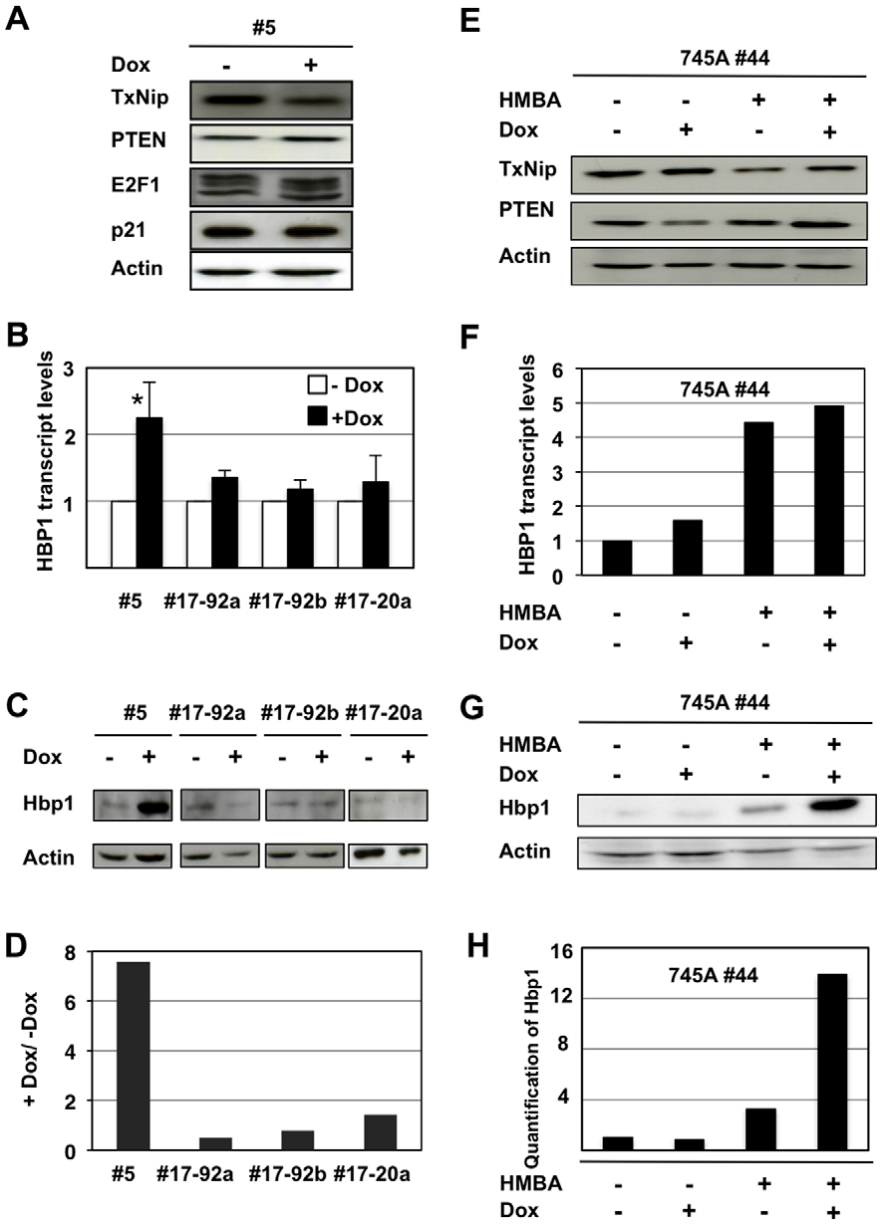
miR-17 and miR-20a contribute to Hbp1 regulation in NN10#5 and 7451#44 cells. A, B, C and D : Parental NN10#5 cells and their derivative #17-92a, #17-92b and #17-20 were cultured for two days in the presence or absence of Dox before Western blot and qRT-PCR analyses of known targets of miR-17-92 cluster. A: Western blot analysis of non-regulated targets in NN10#5 cells. B: Histograms show relative levels of hbp1 mRNA in the indicated Dox-treated cells normalized to that in untreated cells (means and standard deviations from 3 experiments). Asterisks indicate significant differences induced by Dox treatment (p<0.05). C: Western blot analysis of Hbp1 protein. D: Quantification of Hbp1 protein level variations in response to Dox between the indicated cells (+Dox/-Dox ratios of Hbp1 signal standardized to Actin signal determined by densitometric analysis of Western blot shown in C). E, F, G and H : 745#44 cells were grown as in Figure 1 for two days in the presence or absence of HMBA or Dox as indicated. E: Western blot analysis of non-regulated targets. F: Histograms show relative levels of hbp1 mRNA (typical results from two different experiments). G: Western blot analysis of Hbp1 protein (typical results from two different experiments). H: Quantification of relative levels of Hbp1 protein standardized to actin.

Having established miR-17 and miR-20a functionality in NN10#5 cells proliferation downstream of Fli-1, we tried to identify miR-17/20a targets able to explain this effect. Surprisingly, Western blots analyses revealed that none of several miR-17/miR-20a targets already identified in other cell contexts (including p21, PTEN, E2F1 or TXNIP) displayed the expected increased level in response to Dox treatment (Figure 7A). We then looked for other possible candidates in the transcriptome analysis presented in Figure 3. We noticed that HBP1, which has been identified as a miR-17 target in human breast cancer cells [37] was included in the leading edge genes subset whose expression increases in response to HMBA in 745A#44 cells. Interestingly, HBP1 mRNA levels also increased in response to Dox in NN10#5 cells and importantly this increase was strongly attenuated in #17-92a or #17-20a cells (Figure 7B). Western blots analyses showed that HBP1 protein levels strongly increased in response to Dox in NN10#5 cells but not in the #17-92a, #17-92b or #17-20a clones (Figure 7C). Moreover, the >7 fold HBP1 protein level increase in Dox-treated parental NN10#5 cells was much higher than the 2 fold mRNA level increase (Figure 7D), as expected from the known miRNA mechanism of action at the translation level. Similarly, we found no variation of TXNIP or PTEN protein levels in 745A#44 cells in response to HMBA or Dox treatment (Figure 7E). In contrast, both HBP1 transcript (Figure 7F) and HBP1 protein (Figure 7G, H) levels were strongly increased in response to HMBA while HBP1 protein levels but not HBP1 mRNA were further markedly increased in response to Dox thus indicating a post-transcriptional regulation. From these results, we conclude that HBP1 can be considered as an authentic miR-17 and miR-20a target in both NN10#5 and 745A#44 cells. These results prompted us to try to restore the proliferation of NN10#5 cells in the presence of Dox using Hbp1 siRNA transfection. Unfortunately, despite successful knockdown of Hbp1 mRNA and slight but significant increase of cell proliferation, Hbp1 siRNA transfection was not associated with concomitant decrease of Hbp1 protein level thus alleviating definitive conclusion concerning the real contribution of Hbp1 downstream of Fli-1 and Fli-3 (Figure S2).

## Discussion

Friend erythroleukemia develops in susceptible mice infected by the two virus of the Friend viral complex: SFFV and F-MuLV. Although proviral insertions are supposed to randomly occur into infected cells genome, clonal erythroleukemia only develops from erythroid progenitors harboring proviral insertion at one of a very few loci, often leading to neighboring genes constitutive activation. More than 75% of these SFFV or F-MuLV recurrent proviral insertions occur upstream of *spi-1* or *fli-1* genes, respectively, and lead to the constitutive expression of the ETS family transcription factors Spi-1 and Fli-1, which play a major role in blocking differentiation and promoting proliferation and cell survival. Such highly recurrent activation of one of these two transcription factors belonging to the same ETS family led us to hypothesize that Spi-1 and Fli-1 could contribute to erythroleukemia through common target genes deregulation. In strong support to this hypothesis, we recently established that Spi-1 and Fli-1 indeed directly activate many common target genes involved in ribosome biogenesis [18] and do contribute to high ribosomes content in Friend erythroleukemic cells (data not shown). Going one step further, we showed here that Spi-1 and Fli-1 are also direct activators of the miR-17-92 miRNA cluster, previously identified as the third most frequently activated oncogenic locus by F-MuLV proviral insertions. Moreover, we showed that restoration to their initial levels of only two members of this cluster, miR-17 and miR-20a, is sufficient to partially rescue the loss of proliferation induced by Fli-1 knock-down in erythroleukemic cells harboring activated *fli-1* locus. Altogether, this study allows us to conclude that three of the most recurrently activated oncogenes in Friend erythroleukemia contribute to a same oncogenic network required to promote erythroleukemic cells proliferation.

Our experimental results support a direct activation of miR-17-92 cluster by Spi-1 and Fli-1 based on the strong correlation between pri-miR-17-92 and Spi-1 and/or Fli-1 levels, on the dependency of 40% of miR-17-92 promoter activity on the integrity of a conserved ETS binding site and on the *in vivo* recruitment of Spi-1 and/or Fli-1 on the corresponding region in both 745#44 and NN10#5 erythroleukemic cells. Knowing that Myc is a major positive regulator of miR-17-92, we also excluded its contribution downstream of Spi-1 and Fli-1 by verifying that Myc protein levels were not affected by Spi-1 and/or Fli-1 knockdown in 745#44 or by Fli-1 knockdown in NN10#5 cells (Figure S3). In contrast to the present results obtained in erythroleukemic cells, another recent study showed that Spi-1/PU.1 increase involved in macrophage differentiation contributes to miR-17-92 down-regulation without PU.1 binding on its promoter but through the direct activation of *egr2* gene encoding a transcriptional repressor of miR-17-92 promoter activity [38]. These different results suggest that miR-17-92 regulation by Spi-1/PU.1 is strongly cell context dependent.

Our experimental results support the functional implication of miR-17 and miR-20a in erythroleukemic cells proliferation downstream of Fli-1 based on the rescue of proliferation loss induced by Fli-1 knock down following the restoration of these two miRNA to their initial levels. Moreover, transfection of miR-17 and miR-20a anti-miRs confirmed that this rescue is not due to clone effect and further supports our conclusion. This conclusion is in apparent contradiction to the recent demonstration of the anti-proliferative effect of miR-17 in normal erythroid progenitors by Li et al [29]. However, interesting comparison between the two studies may help to understand the basis of these opposite effects of miR-17 on cell proliferation. First, Li et al. achieved robust miR-17 overexpression by retroviral transduction. Since it seems reasonable to think that miR-17 effect could be dose-dependent, it would be interesting to compare miR-17 levels between NN10#5 cells and cell lines overexpressing it from Li et al. study. We noticed that miR-17 levels in NN10#5 cells are notably low compared to K16 erythroleukemic cells harboring a *fli-3* rearrangement [5] (Figure S4). One possibility could be that miR-17 levels in NN10#5 cells are insufficient to reduce proliferation as it is observed following strong overexpression in erythroid progenitors [29]. We also noticed that the antiproliferative effect of miR-17 was associated with increased levels of P53, thus suggesting [29] that P53 might be functionally involved. The absence of functional P53 in NN10 (F Moreau-Gachelin, personal communication) and 745A cell lines [39] might therefore be another cause of the lack of antiproliferative effect of miR-17 in these cell lines. In that context, the identification of Hbp1 as a miR-17 target in NN10#5 and 745#44 cells is interesting. Indeed, in addition to being a negative regulator of the Wnt signaling pathway [40] or of MIF [41], Hbp1 is known to interact with Myc and to inhibit its transcriptional activity [42]. Moreover, Myc itself is a well known oncogene that stimulates proliferation but can also induce P53-dependent proliferation arrest due to its capacity to activate the ARF-MDM2-P53 oncogenic stress pathway when expressed at high levels [43]. Altogether these data lead us to propose a working model where the duality of miR-17 effect on cell proliferation could be explained by its dose-dependent and Hbp1-mediated regulation of Myc activity and its strong dependence on the p53 status of the cells (see diagram in Figure S5).

During this study, we found that several other known miR-17-92 targets such as PTEN (miR-19 and miR-17/20a target) [24], TxNIP/VDUP (miR-17/20a target) [44] E2F1 (miR-17/20a) [45], [46] and P21 (miR-17/20a target) [45], [47] were surprisingly not regulated in response to miR-17-92 expression variations in NN10#5 and 745A#44 cells. These results highlight the increasingly recognized cellular context dependency of miRNA activity. For example, several RNA-binding proteins have been recently shown to control the accessibility and regulation of a given mRNA by a specific miRNA such as PUM2 that is involved in p27 mRNA regulation by miR-221 and miR-222 [48] or RPL11 involved in the control of Myc mRNA regulation by miR-24 [49]. Furthermore, target mRNA regulation by a given miRNA is dependent on the total amount of all other mRNA expressed in the same cell that are able to bind this given miRNA and thus to act as competitors [50]. The identification of such missing putative RNA binding proteins and/or excess of mRNA competitors responsible for the lack of regulation of several known miR-17-92 targets in NN10 erythroleukemic cells remains an interesting challenge for future studies.

Interestingly, deregulation of ETS transcription factors and miR-17-92 was independently described in multiple other proliferative contexts. For example, Fli-1 levels are known to be involved in the proliferation control of activated splenic B lymphocytes [51] whereas deregulated miR-17-92 has been independently involved in lymphoid hyperproliferative diseases [20]. Furthermore, it was shown that B-lymphocytes from human lupus patients [52] or from lupus erythematous mouse model MRL/lrp displayed increased Fli-1 expression [53] whereas another study showed an up-regulated miR-17-92 expression in mouse models of lupus [54]. It is therefore very tempting to speculate that the Fli-1/miR-17-92 cascade identified here in mouse erythroleukemic cells could also be involved in stimulation of normal and pathological B cell proliferation. Similarly, Fli-1 has been shown to contribute to the high malignancy of the breast cancer cell line MDA-MB231 [55] whereas another study independently showed the inhibition of Hbp1 by miR-17 in the same cells [37]. Moreover, miR-20a has been shown to targets E2F1 and prevents apoptosis of the PC3 prostate cancer cell line [46] whose tumorigenicity has been independently shown to be dependent on Ets2 transcription factor [56] while another prostate cell line is also characterized by Ets2 over expression and Hbp1 down regulation [41]. These other data further suggest that miR-17-92 deregulation could be a generic property of several other ETS factors contributing to their oncogenicity.

In conclusion, the present study demonstrated that the three most frequently activated oncogenes in Friend erythroleukemia are involved in a same oncogenic network including an ETS/miR-17-92 module that might be involved in other proliferative contexts.

## Supporting Information

Data S1 **Microarray data.** Microarray data from 745#44 cells untreated (−H−D) or treated for two days in the presence of Dox alone (−H−D), HMBA alone (+H−D) or both Dox and HMBA (+H+D) have been previously obtained (Juban et al, 2009, Mol Cell Biol 29: 2852–64). These microarray data are included in the following three files: Manip745.gct (normalized expression data); Manip745.chip (microarray probe description); Manip745.cls (class description). These three files are provided as a single compressed file «microarray data.zip».
(ZIP)

Data S2 **GSEA analyses.** Lists of predicted miRNA targets were established from the miRWalk database (http://www.umm.uni-heidelberg.de/apps/zmf/mirwalk/) by compiling all mouse transcripts with 3′UTR harboring putative target sequences predicted by the three different algorithms miRanda, miRDB and miRWalk with a p value<0.05. Predicted target gene lists were then used to perform gene set enrichment analyses using the GSEA software v2.0 (http://www.broadinstitute.org/gsea/index.jsp/) after uploading the above three microarray data files and performing two successive comparisons (−H−D vs +H+D and −H−D vs +H−D). Gene list results are given on separate sheets for each miRNA in file Data S2 (“Gene Lists GSEA.xlsx”): Column 1: predicted target gene list used for GSEA; Column 2: subset list of predicted target genes present on microaarray; Column 3: leading edge subset of genes that were found to be either up or downregulated by comparing −H−D vs +H+D (normalized p value indicated on the top of the column); Column 4: leading edge subset gene list that were found to be either up or downregulated by comparing −H−D vs +H+D (normalized p value indicated on the top of the column); Column 5: intersection between leading edge gene lists in columns 3 and 4. Lists of leading edge common targets for miR-17 and miR-20 (intersection of the four gene lists in columns 3 and 4 on sheets miR-17 and miR-20), miR-19a and miR19b (intersection of the four gene lists in columns 3 and 4 on sheets miR-19a and miR-19b) as well as for miR-451 (intersection of gene lists in columns 3 and 4 in sheet miR-451) that have been used to generate the histograms presented in Figure 3B, C and D are given in sheet named «Gene list profile».
(XLSX)

Figure S1 **Restoration of pri-miR-17-92 and miR-20a levels in 745#44 cells does not rescue the decrease of cell proliferation induced by Fli-1 loss.** Clone 745#44G3 was derived from 745#44 cells following transfection with plasmid pcDNA4/17-92 carrying the whole miR-17-92 cluster under the control of a Dox-inducible promoter. Equal number of 745#44 and 745#44G3 cells were then seeded in the presence or absence of Dox, numbered every next three days whereas both pri-miR-17-92 and miR-20a levels were quantified at day 2 by qRT-PCR as in Figure 1A. A: Results of qRT-PCR showing the rescue of both pri-miR-17-92 and miR-20a levels in the presence of Dox in 745#44G3 cells. B: Results of cells numbering showing the same decrease of proliferation induced by Dox treatment in both 745#44G3 and 745#44 cells.
(TIF)

Figure S2 **Hbp1 siRNA transfection increases NN10#5 cells proliferation in the presence of Dox.** Dox-treated NN10#5 cells were transfected twice either with Hbp1 siRNA or with control Luc siRNA 24 h and 48 h following the first addition of Dox and then analyzed 24 h later. A: relative levels of Hbp1 mRNA (standardized to actin mRNA) determined by qRT-PCR and showing expected decrease following Hbp1 siRNA transfection compared to control siRNA. B: Western blot analysis of Hbp1 and actin (loading control) proteins showing no detectable decrease following Hbp1 siRNA transfection compared to control siRNA. C: final cell concentration (mean and standard deviation from 3 independent experiments) showing significant increase induced by Hbp1 siRNA transfection compared to control Luc siRNA. Note that the discrepancy between the expected decrease of Hbp1 mRNA but the absence of corresponding decrease of Hbp1 proteins levels might reflect complex post-transcriptional and/or post-translational regulations but does not formally exclude the occurrence of transient Hbp1 protein levels induced by Hbp1 siRNA during the course of the experiment. However due to this discrepancy no definitive conclusion could be drawn concerning the real contribution of the increase of Hbp1 to proliferation arrest induced in the presence of Dox.
(TIF)

Figure S3 **Western-blot analysis of Myc proteins in treated and untreated 745#44 and NN10#5 cells.** 745#44 cells (A) were cultured for two days in the presence or absence of HMBA and/or Dox as in Figure 1A and NN10#5 for two days in the presence or absence of Dox as in Figure 3A before Western blot analysis of Myc and actin (loading control) proteins. The absence of detectable variation of Myc proteins levels indicates that Myc does not contribute to the decrease of miR-17-92 miRNA cluster expression observed in treated cells (see Figure 1A and 3A).
(TIF)

Figure S4 **Comparison of miR-17, miR18, miR-19a, miR19b and miR92 levels between NN10#5, 745A#44 and K16 erythroleukemic cells.** miRNA levels were determined by q-RT-PCR and standardized to that of U6 RNA (means and standard deviations from 3 independent experiments). Note that these quantifications are not absolute quantifications and only allow confident comparisons for each given between the different cells lines. Variations of the miRNA/U6 ratios between miRNAs are only indicative of the real relative levels of the corresponding miRNAs assuming a same efficiency for all PCR reactions. Significant differences between cell lines are indicated by asterisks. Table shown under the histogram indicate Student tests p-values obtained in two by two comparisons performed between cell lines for each miRNA.
(TIF)

Figure S5 **Diagram summarizing the inter-relationships established in our study and illustrating the pro-proliferative (A) or anti-proliferative (B) alternative contribution of miR-17-20a.** Previously known and newly established relationships are indicated in black and red respectively. Myc has already been shown to either stimulate proliferation (oncogenic pathway 1) when expressed at low levels or to induce proliferation arrest through the ARF-MDM2-P53 pathway (oncogenic stress pathway 2) when expressed at high levels. HBP1 itself has already been shown to interact and to inhibit Myc activity. Due to this property of HBP1, our finding that miR17 and miR20a downregulate HBP1 strongly suggests that these two miRNA contribute to increase Myc activity. In fully transformed cells with no functional P53 such as established erythroleukemic cells lines (A) increased Myc activity induced by deregulated expression of miR17 and miR20a cannot induce oncogenic stress thus explaining their pro-proliferative effect. In contrast, in cells harboring functional P53 such as normal erythroid progenitors (B), increased Myc activity mediated by deregulated expression of miR17 and miR20a induces oncogenic stress thus explaining their anti-proliferative effect. Please note that independently of the Spi-1->Fli-1>Fli-3 regulatory cascade, Spi-1, Fli-1 and Fli-3 activations obviously each display parallel and specific contributions that, for clarity, are not presented on this simplified diagram. The existence of these parallel contributions is clearly indicated by our finding that complete restoration of Fli-3 levels only partially rescues proliferation loss following Fli-1 knockdown in NN10#5 cells (see Figure 6A) and does not rescue proliferation loss following Fli-1 knockdown in 745#44 cells (see Figure S1).
(TIF)

Table S1 **Oligonucleotides used in this study.**
(DOCX)
