# Supplementary figures and images for: Spi-1, Fli-1 and Fli-3 (miR-17-92) Oncogenes Contribute to a Single Oncogenic Network Controlling Cell Proliferation in Friend Erythroleukemia
Source: PLoS One. 2012 Oct 8;7(10):e46799. doi: 10.1371/journal.pone.0046799 (PMC3466182; doi:10.1371/journal.pone.0046799)

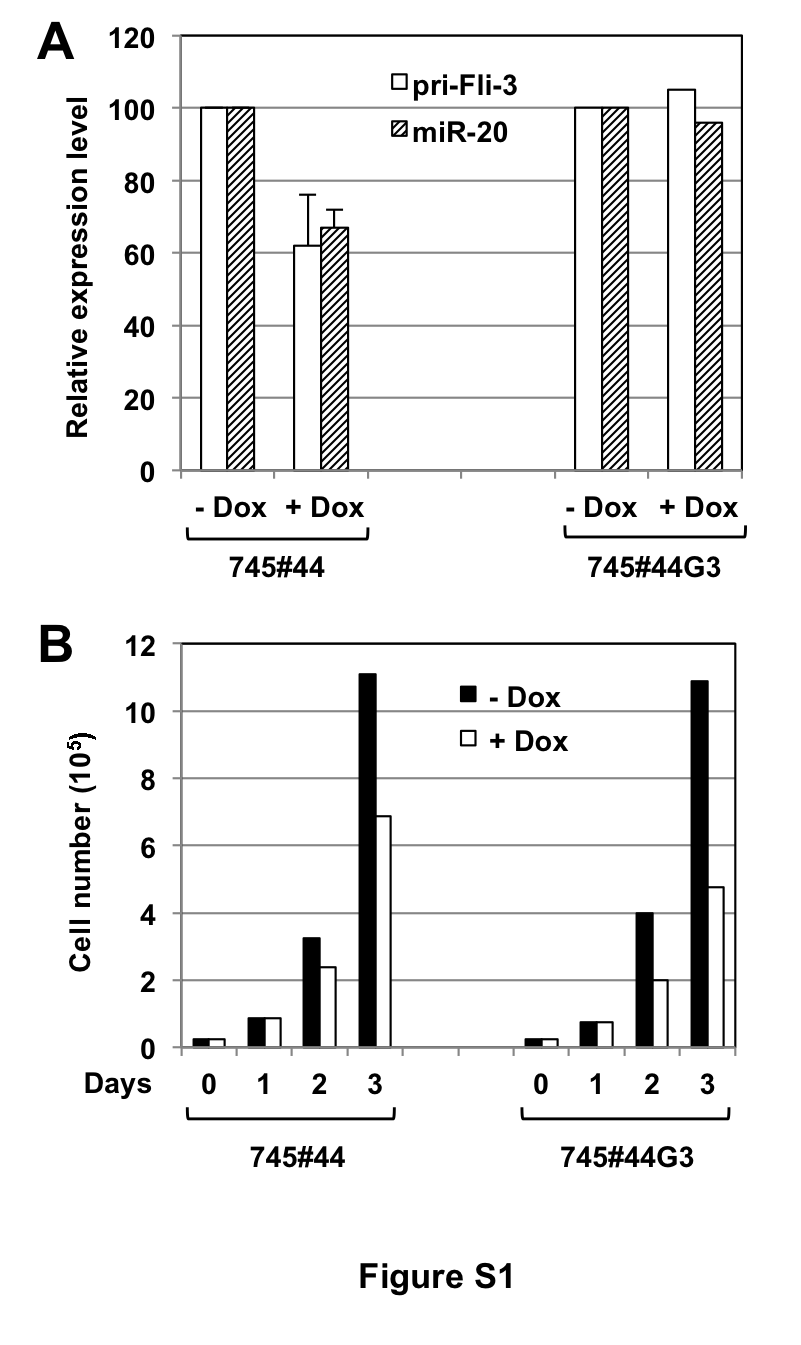

Supplement: Figure S1 — Restoration of pri-miR-17-92 and miR-20a levels in 745#44 cells does not rescue the decrease of cell proliferation induced by Fli-1 loss. Clone 745#44G3 was derived from 745#44 cells following transfection with plasmid pcDNA4/17-92 carrying the whole miR-17-92 cluster under the control of a Dox-inducible promoter. Equal number of 745#44 and 745#44G3 cells were then seeded in the presence or absence of Dox, numbered every next three days whereas both pri-miR-17-92 and miR-20a levels were quantified at day 2 by qRT-PCR as in Figure 1A. A: Results of qRT-PCR showing the rescue of both pri-miR-17-92 and miR-20a levels in the presence of Dox in 745#44G3 cells. B: Results of cells numbering showing the same decrease of proliferation induced by Dox treatment in both 745#44G3 and 745#44 cells. (TIF) [file pone.0046799.s003.tif]

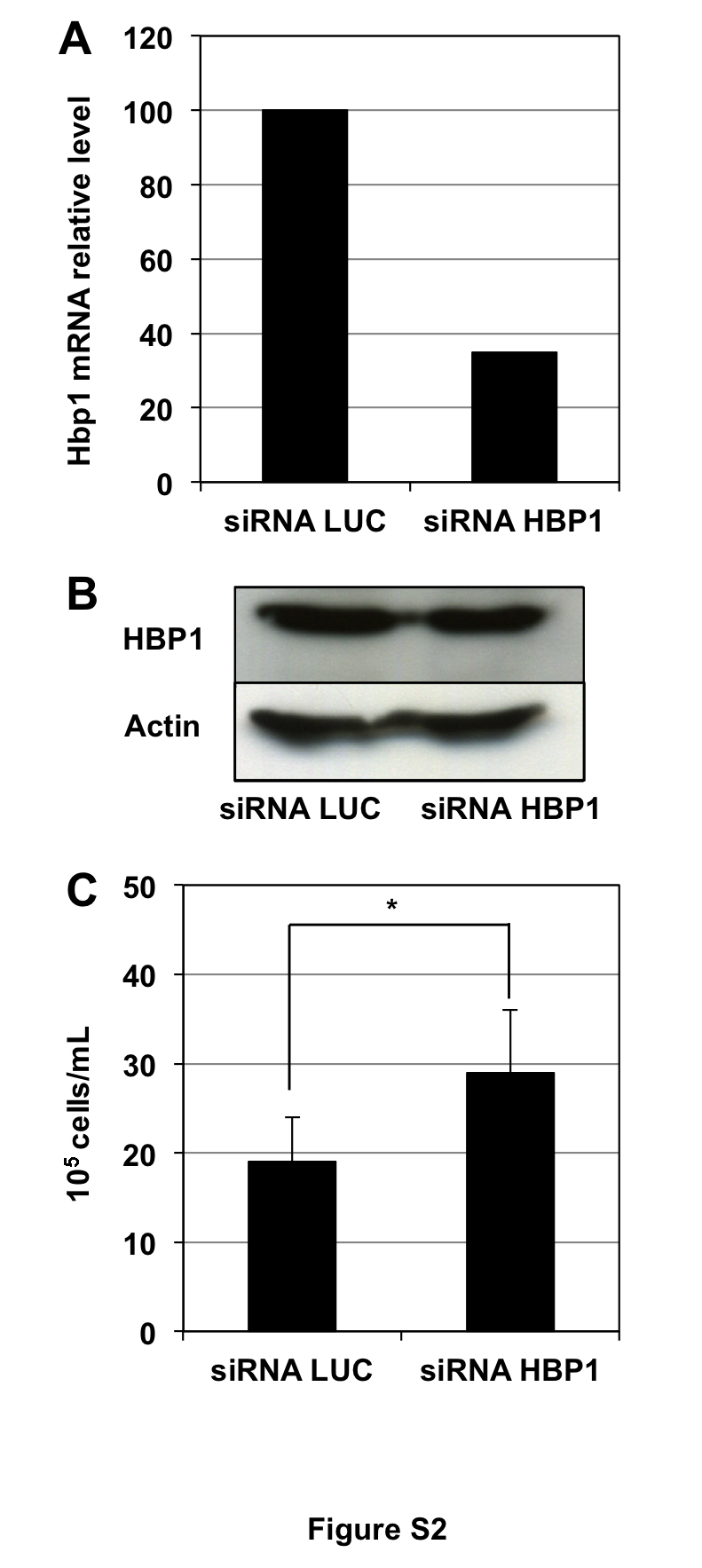

Supplement: Figure S2 — Hbp1 siRNA transfection increases NN10#5 cells proliferation in the presence of Dox. Dox-treated NN10#5 cells were transfected twice either with Hbp1 siRNA or with control Luc siRNA 24 h and 48 h following the first addition of Dox and then analyzed 24 h later. A: relative levels of Hbp1 mRNA (standardized to actin mRNA) determined by qRT-PCR and showing expected decrease following Hbp1 siRNA transfection compared to control siRNA. B: Western blot analysis of Hbp1 and actin (loading control) proteins showing no detectable decrease following Hbp1 siRNA transfection compared to control siRNA. C: final cell concentration (mean and standard deviation from 3 independent experiments) showing significant increase induced by Hbp1 siRNA transfection compared to control Luc siRNA. Note that the discrepancy between the expected decrease of Hbp1 mRNA but the absence of corresponding decrease of Hbp1 proteins levels might reflect complex post-transcriptional and/or post-translational regulations but does not formally exclude the occurrence of transient Hbp1 protein levels induced by Hbp1 siRNA during the course of the experiment. However due to this discrepancy no definitive conclusion could be drawn concerning the real contribution of the increase of Hbp1 to proliferation arrest induced in the presence of Dox. (TIF) [file pone.0046799.s004.tif]

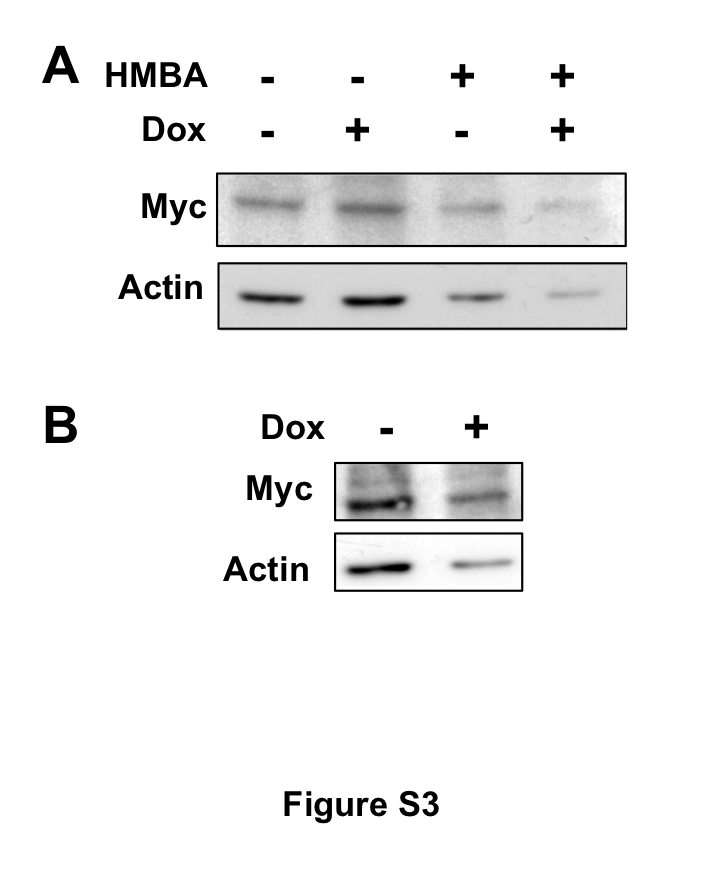

Supplement: Figure S3 — Western-blot analysis of Myc proteins in treated and untreated 745#44 and NN10#5 cells. 745#44 cells (A) were cultured for two days in the presence or absence of HMBA and/or Dox as in Figure 1A and NN10#5 for two days in the presence or absence of Dox as in Figure 3A before Western blot analysis of Myc and actin (loading control) proteins. The absence of detectable variation of Myc proteins levels indicates that Myc does not contribute to the decrease of miR-17-92 miRNA cluster expression observed in treated cells (see Figure 1A and 3A). (TIF) [file pone.0046799.s005.tif]

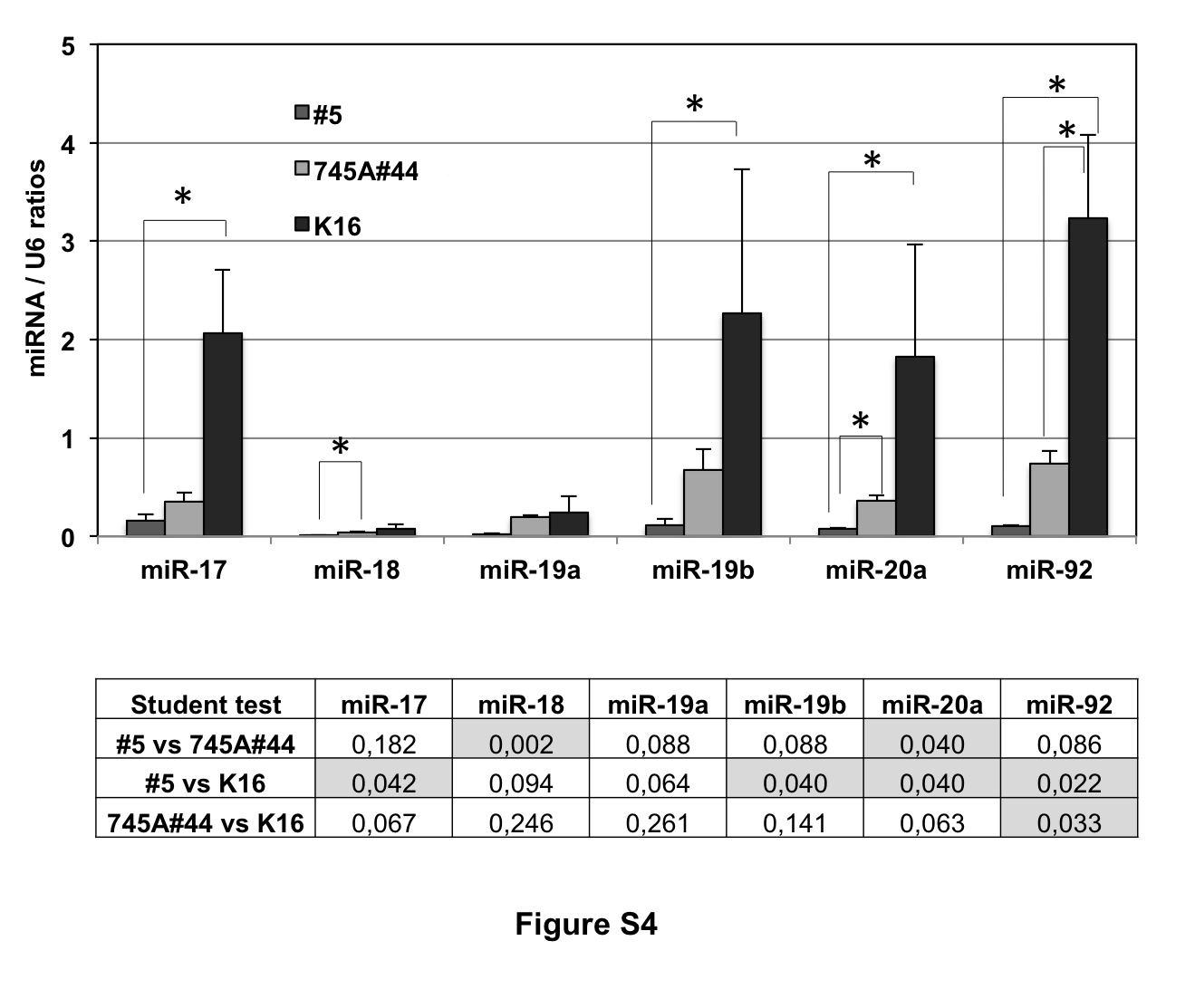

Supplement: Figure S4 — Comparison of miR-17, miR18, miR-19a, miR19b and miR92 levels between NN10#5, 745A#44 and K16 erythroleukemic cells. miRNA levels were determined by q-RT-PCR and standardized to that of U6 RNA (means and standard deviations from 3 independent experiments). Note that these quantifications are not absolute quantifications and only allow confident comparisons for each given between the different cells lines. Variations of the miRNA/U6 ratios between miRNAs are only indicative of the real relative levels of the corresponding miRNAs assuming a same efficiency for all PCR reactions. Significant differences between cell lines are indicated by asterisks. Table shown under the histogram indicate Student tests p-values obtained in two by two comparisons performed between cell lines for each miRNA. (TIF) [file pone.0046799.s006.tif]

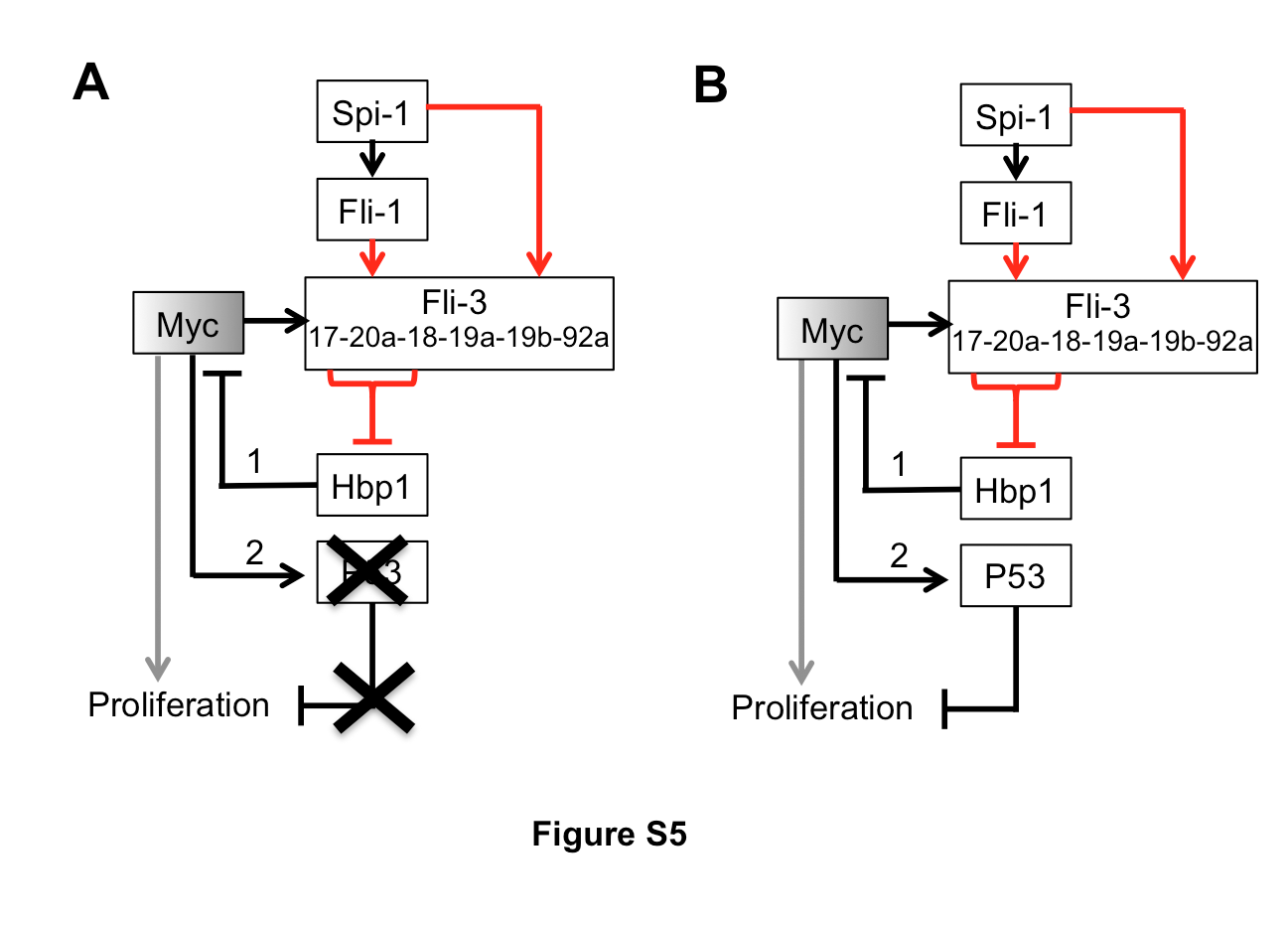

Supplement: Figure S5 — Diagram summarizing the inter-relationships established in our study and illustrating the pro-proliferative (A) or anti-proliferative (B) alternative contribution of miR-17-20a. Previously known and newly established relationships are indicated in black and red respectively. Myc has already been shown to either stimulate proliferation (oncogenic pathway 1) when expressed at low levels or to induce proliferation arrest through the ARF-MDM2-P53 pathway (oncogenic stress pathway 2) when expressed at high levels. HBP1 itself has already been shown to interact and to inhibit Myc activity. Due to this property of HBP1, our finding that miR17 and miR20a downregulate HBP1 strongly suggests that these two miRNA contribute to increase Myc activity. In fully transformed cells with no functional P53 such as established erythroleukemic cells lines (A) increased Myc activity induced by deregulated expression of miR17 and miR20a cannot induce oncogenic stress thus explaining their pro-proliferative effect. In contrast, in cells harboring functional P53 such as normal erythroid progenitors (B), increased Myc activity mediated by deregulated expression of miR17 and miR20a induces oncogenic stress thus explaining their anti-proliferative effect. Please note that independently of the Spi-1->Fli-1>Fli-3 regulatory cascade, Spi-1, Fli-1 and Fli-3 activations obviously each display parallel and specific contributions that, for clarity, are not presented on this simplified diagram. The existence of these parallel contributions is clearly indicated by our finding that complete restoration of Fli-3 levels only partially rescues proliferation loss following Fli-1 knockdown in NN10#5 cells (see Figure 6A) and does not rescue proliferation loss following Fli-1 knockdown in 745#44 cells (see Figure S1). (TIF) [file pone.0046799.s007.tif]
