# Supplementary material for: Spi-1, Fli-1 and Fli-3 (miR-17-92) Oncogenes Contribute to a Single Oncogenic Network Controlling Cell Proliferation in Friend Erythroleukemia
Source: PLoS One. 2012 Oct 8;7(10):e46799. doi: 10.1371/journal.pone.0046799 (PMC3466182; doi:10.1371/journal.pone.0046799)
Supplement: Table S1 — Oligonucleotides used in this study. (DOCX) [file pone.0046799.s008.docx]

**Supplementary table 1**

Oligonucleotides sequences

| **Cloning primers** | |
| --- | --- |
| miR-17-92 F (HindIII) | GAG AGA AAG CTT TTT GGA ACT TCT GGC TAT TGG C |
| miR-17-92 R (XhoI) | AG AGA CTC GAG AAA TCC AGC GAG CAA ACA GC |
| miR-17 R (EcoRI) | GAG AGA GAA TTC AAA AAG CAC GCA GCA CCA GC |
| miR-20a F (EcoRI) | GAG AGA GAA TTC TTG TGT CGA TGT AGA GCC TGC G |
| miR-20a R (XhoI) | GAG AGA CTC GAG CCA TAG ACC AGT GCT CAA TAA CAG G |
| -645/+15 promoter F | GAG AGA CTC GAG ACT TCT CGG TGC TGC ACT AGG |
| -645/+15 promoter R | GAG AGA CTC GAG GAC CAT GTG GGT GAA TGA AG |
| -78 EBS mutagenesis DE | GAG AGA ACG CGT TAC TTT GTT TTT TAT GCT AAT GAG AGG AG |
| -78 EBS mutagenesis UE | GAG AGA ACG CGT ACC TCC CCG CCC GCC CGC CAA TC |
| **qRT-PCR primers (mRNA and pri-miRNA quantification)** | |
| HBP1 F | AGC CAT AAG TGT GAT CCT TG |
| HBP1 R | GCA GAC TTA CAA ACA CAA GC |
| Pri-miR-17-92 F | CAA AGT GCT TAC AGT GCA GGT AG |
| Pri-miR-17-92 R | TAT CTG CAC TAG ATG CAC CTT A |
| Beta-Actine F | TGG GGA ATG GGT CAG AAG GAC TC |
| Beta-Actine R | TGG GGA ATG GGT CAG AAG GAC TC |
| **qPCR primers (ChIP assays)** | |
| mir-17-92 EBS F | TAA TGA GGG AGTGGG GCT TGT C |
| mir-17-92 EBS R | CGA AGG ACC ATG TGG GTG AAT G |
| Primers used for Gapdh promoter and HS129k (mouse α-globin locus) have been published in Anguita et al. EMBO J. 2004 23(14) 2841-52. | |
| **siRNA** | |
| Hbp1 siRNA (antisense) | AUU UUU UUC CAC CUG UCA C |
| Luc siRNA (antisense) | UCG AAG UAU UCC GCG UAC G |
